# Exploring a Unique Case of Hailey-Hailey Disease: Understanding the Manifestation of Fragile Skin

**DOI:** 10.7759/cureus.69432

**Published:** 2024-09-14

**Authors:** Lakshmi Priya Asokan, Sumithra Arumugam, Sulochana Sonti

**Affiliations:** 1 Department of Pathology, Saveetha Medical College and Hospital, Saveetha Institute of Medical and Technical Sciences, Saveetha University, Chennai, IND

**Keywords:** acantholysis, autosomal dominant, hailey-hailey disease, intraepidermal blister, pemphigus

## Abstract

Hailey-Hailey disease (HHD) is a rare autosomal dominant chronic blistering disorder. It presents with eruption of small vesicles, erosions, and crusted plaques frequently in the intertriginous areas. Family history is present in some cases. Histopathology plays a crucial role in diagnosing HHD, which is identified by its distinct "dilapidated brick wall" appearance. Due to its uncommon nature and similarity to other skin conditions in intertriginous areas, HHD can often be misdiagnosed by clinicians. We present a case of a 46-year-old female with crusted lesions in the neck, antecubital fossa, submammary folds, and inguinal regions. There were recurrent exacerbations and remissions. Biopsy was sent for histopathological examination, which helped in confirming the diagnosis of HHD.

## Introduction

Hailey-Hailey disease (HHD) was described for the first time by the Hailey brothers in 1939 [1]. It is also known as familial benign pemphigus and is a chronic intraepidermal blistering disorder. It is a relatively rare condition with an incidence of 1:50,000 [2]. It is inherited as an autosomal dominant trait due to a mutation in the ATP2C1 gene [3]. About two-thirds of the cases have a positive family history. It is commonly found in teenagers and young adults, usually at or after puberty. It presents with the eruption of small vesicles, erosions, and crusted plaques frequently in the intertriginous areas [3]. The hypothesis that defective junctional complexes in keratinocytes cause the structurally weak epidermis seen in HHD is supported by two key features: skin lesions in trauma-prone areas, where lesions typically appear in the areas of the skin that are subject to physical stress or friction, suggesting that the epidermis is particularly vulnerable in these regions. In affected epithelial tissue, there is acantholysis, which is the loss of connections between keratinocytes, leading to the separation of these cells. However, this occurs without the actual rupture of the keratinocytes themselves, indicating that the junctions between cells are compromised. These points imply that the weakened junctional complexes between keratinocytes in HHD make the skin structurally fragile, leading to the characteristic lesions observed in the disease [4]. The predilection to intertriginous areas in developing these erosions may be due to heat, moisture, and friction. Histopathology typically displays distinctive characteristics and frequently serves to confirm the diagnosis. Superinfection with bacteria and Candida is commonly observed. It has a chronic course with periods of remissions and exacerbations. Extracutaneous manifestations of this disease are rare [2].

## Case presentation

**Figure 1:**
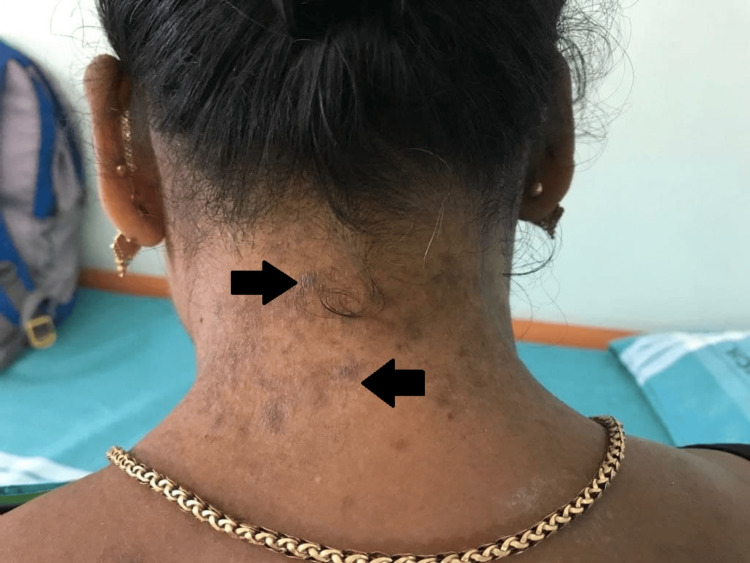
Dark brown patches on the nape of neck (black arrows)

**Figure 2:**
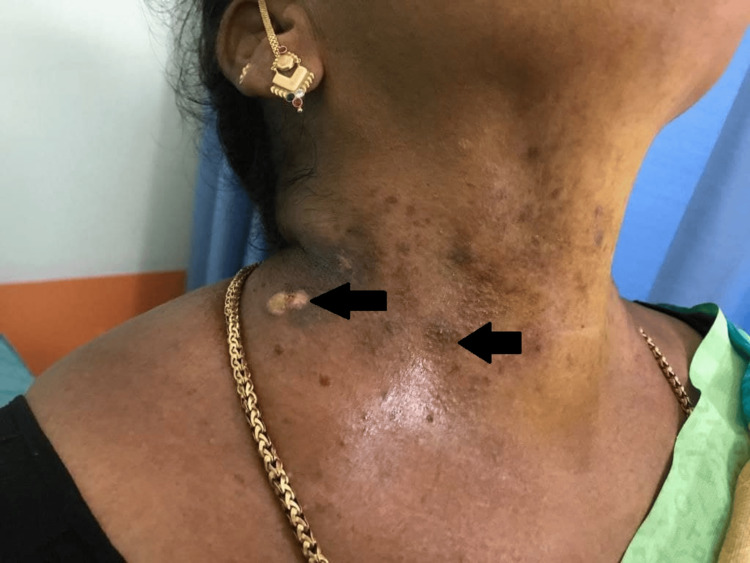
Crusted lesions and erosions on the anterior aspect of the neck (black arrows)

**Figure 3:**
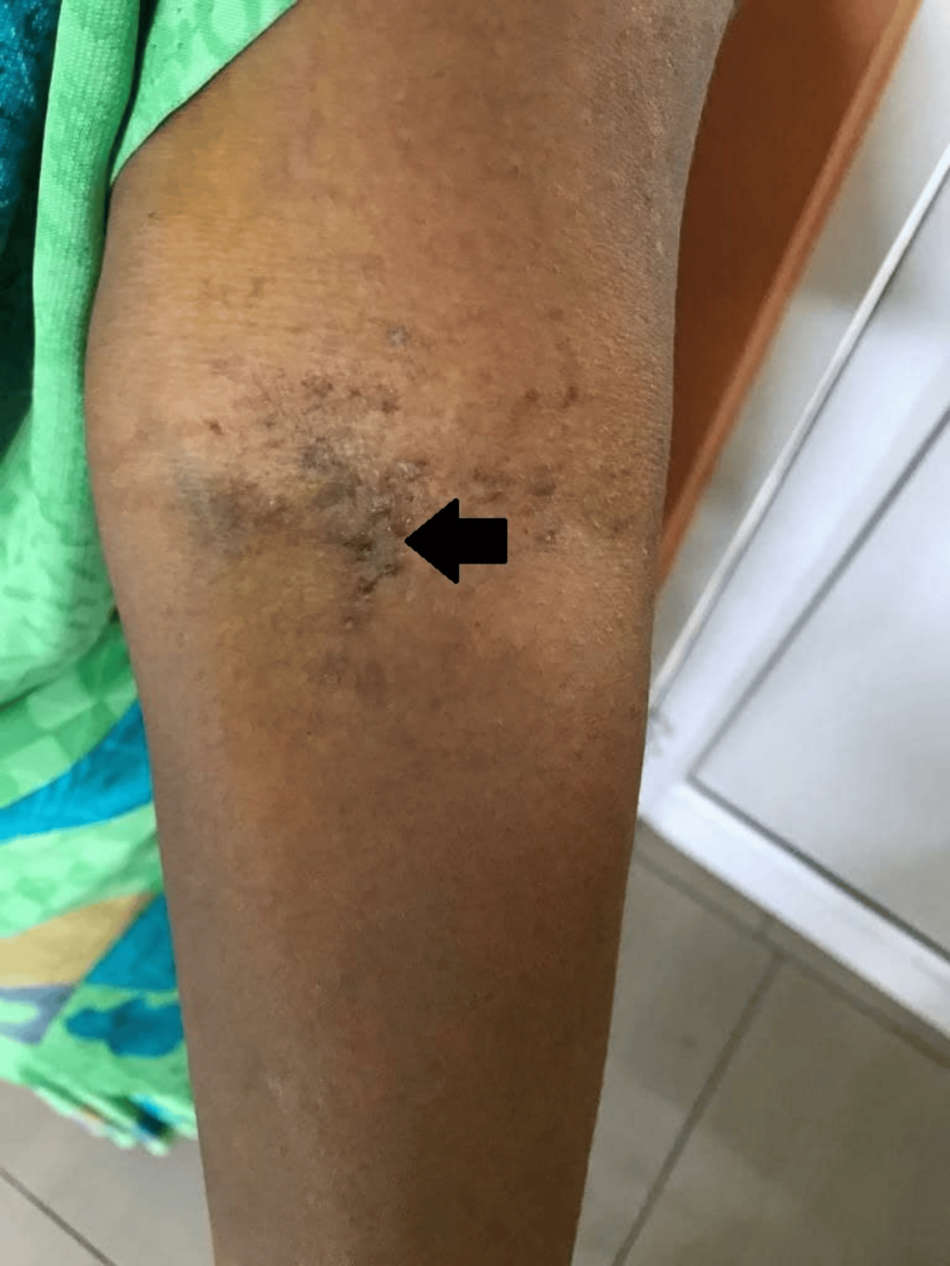
Dark red to brown colored crusted erosions in the antecubital fossa (black arrow)

A 46-year-old female came to the dermatology outpatient department with the complaint of red to dark brown colored crusted lesions. Over the past nine years, she periodically developed erythema and vesicles, which ruptured and formed scabbing skin plaques and crust in the neck, antecubital fossa, submammary folds, and inguinal regions. Lesions were extremely pruritic and associated with a burning sensation. Lesions were gradual in onset with recurrent exacerbations during the summer months. She had a family history of similar complaints in her father and brother. On examination, erythematous crusts and a few vesicles were seen in the neck (Figures 1, 2), antecubital fossa (Figure 3), and submammary folds.

**Figure 4:**
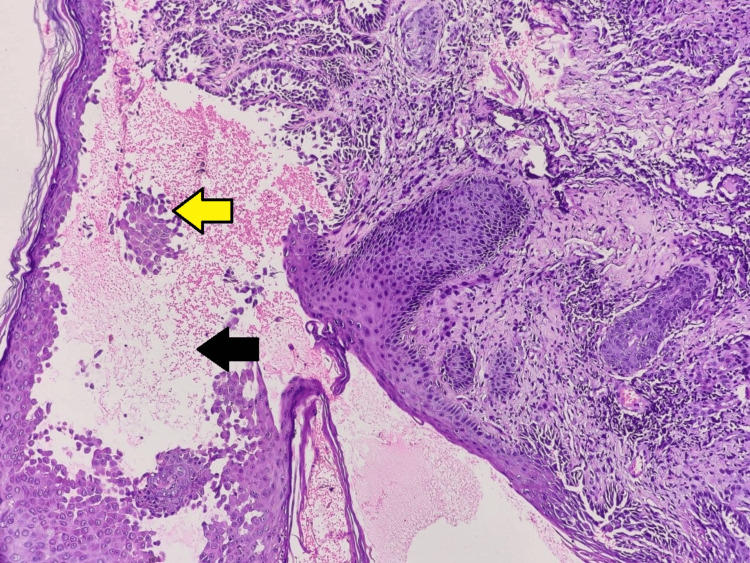
Section of skin showing intraepidermal bullae (black arrow) with acantholysis, depicting dilapidated brick wall appearance (yellow arrow) (hematoxylin and eosin, 200×)

**Figure 5:**
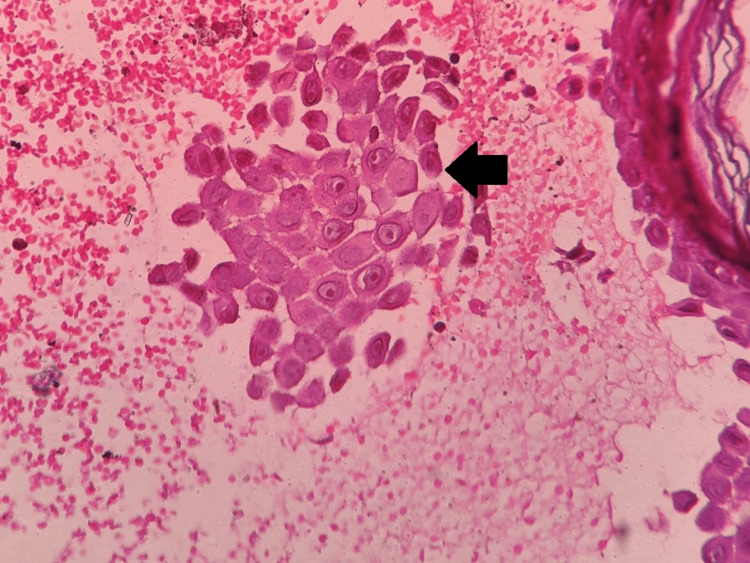
Acantholysis depicting dilapidated brick wall appearance (black arrow) (hematoxylin and eosin, 400×)

**Figure 6:**
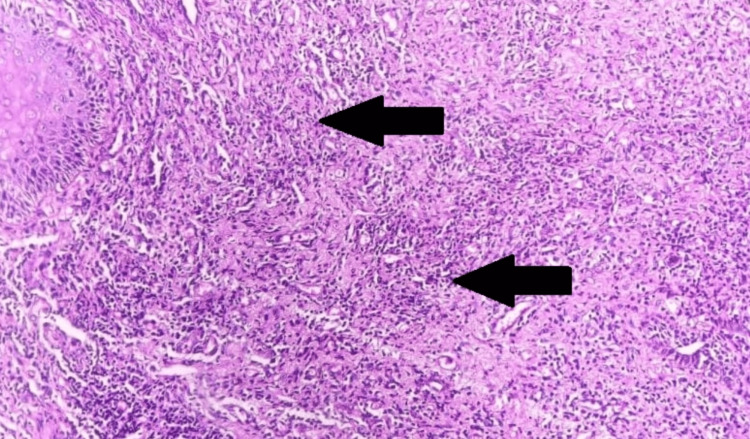
Underlying dermis with lymphoplasmacytic cell infiltrates (black arrows) (hematoxylin and eosin, 200×)

Extracutaneous involvement was not seen. Routine hematological and biochemical investigations were done and were under normal limits. A provisional diagnosis of HHD was made from the clinical presentation and history, so a 3.5 mm punch biopsy was taken from the plaques over the right side of the neck and sent for histopathological examination. On histopathology, the characteristic intraepidermal bullae with acantholysis depict a dilapidated brick wall appearance (Figures 4, 5). The underlying dermis showed lymphoplasmacytic infiltrates (Figure 6), confirming the diagnosis. No evidence of dyskeratosis was observed.

## Discussion

HHD is a rare autosomal dominant disease that occurs in one in 50,000 individuals [2]. The commonly affected age group is between 20 and 30, usually after puberty. No sex predilection is seen. This genodermatosis is due to mutations in the ATP2C1 gene, which encodes the adenosine triphosphatase enzyme [3]. Reduced activity of this enzyme may cause alteration in the intracellular calcium gradient and instability of desmosomal proteins, which are responsible for the adhesion of keratinocytes [4]. This consequently results in acantholysis of the epidermis and vesicle formation. Complete penetrance is seen in adults, but expression is variable in affected families. Two types of mosaicism are observed: type 1: in a patient without evidence of the disease elsewhere, segmental areas as single or multiple localized streaks of disease are seen along Blaschko’s lines. Type 2: severely affected unilateral segmental areas along Blaschko’s lines, which are superimposed on classic disease [5]. HHD clinically presents as grouped erosions and vesicles that rupture easily, leading to dry erythematous, scaly patches, and crusted lesions in the intertriginous areas (neck folds, axillary folds, submammary folds, and inguinal region). With time, patients develop hypertrophic-fissured plaques. Secondary fungal and bacterial superinfections are seen in about 80% of the cases, leading to malodorous or vegetative plaques [6]. HHD is a chronic disease with periods of exacerbations and remissions. Exacerbations can be associated with stress, friction, physical trauma, heat, sweating, and infection. Premenstrual flaring of the disease is seen in some female patients, which suggests some role of sex hormones [7]. Recurrent lesions are often debilitating, both psychologically and physically. Lesions heal without leaving a scar. Usually, extracutaneous involvement is not seen. Rare cases of mucosal involvement, including mouth, esophagus, and labia majora, were reported [8]. On histopathology, suprabasal bulla and acantholysis are seen due to the loss of intercellular bridges. Despite the extensive loss of intercellular bridges, many places show only a slight separation of the detached cells. This is because of the few intact intercellular bridges that hold them loosely together, thus giving the disease's characteristic "dilapidated brick wall" appearance. Adnexal structures are often spared [8]. Direct immunofluorescence is often negative [9]. Histologically, differential diagnosis includes Darier disease and pemphigus vulgaris. Smaller bulla, less pronounced acantholysis, and dyskeratosis with the formation of corps ronds and grains in Darier disease distinguish it from HHD [10]. The presence of eosinophils, less extensive acantholysis, and the absence of dilapidated brick wall appearance distinguish pemphigus vulgaris from HHD [10]. No specific treatment is available for HHD. Symptoms are managed by topical and systemic steroids [11]. Topical antibiotics and antifungals are used to control secondary bacterial and fungal infections. Patients are advised to reduce the triggering factors like heat, friction, and moisture. Wide local excision and replacement with split-thickness skin grafts are done in recalcitrant lesions [12]. CO_2_ laser and erbium-doped yttrium-aluminum-garnet lasers have been reported to be successful [13]. Photodynamic therapy with aminolevulinic acid is effective in some cases [14].

## Conclusions

HHD, though not a life-threatening condition, causes a lot of discomfort to the patients. HHD follows a chronic and fluctuating pattern, with patients experiencing periods of flare-ups and remissions that can last from months to years. While some individuals may notice an improvement in the severity of the disease as they age, others may not see any progress or may even experience worsening of their condition. A detailed family history, complete clinical examination, and histopathology help in establishing the diagnosis of this rare disorder. Topical and systemic antibiotics, corticosteroids, and retinoids can be useful to relieve the symptoms.
